# Diphenhydramine induced acute dystonia: a case report

**DOI:** 10.11604/pamj.2022.42.289.35167

**Published:** 2022-08-17

**Authors:** Danielle Abou Khater, Rafi Daou, Ali Al Dailaty, Mariana Helou

**Affiliations:** 1 Department of Emergency Medicine, Lebanese American University Medical Center, Beirut, Lebanon,; 2 Department of Internal Medicine, Lebanese American University Medical Center, Beirut, Lebanon

**Keywords:** Dystonia, abnormal movements, diphenhydramine, case report

## Abstract

Acute dystonia has notably been a challenge in the emergency unit. Drug-induced dystonia is reported in a limited number of cases in the literature. Rarely, diphenhydramine was found to be the culprit. We report the case of a 25-year-old female patient who developed an acute dystonic reaction following the administration of 25 mg of intravenous diphenhydramine as a treatment for an allergic reaction. The patient was given 5 mg diazepam, admitted for monitoring, and discharged home. Diphenhydramine-induced acute dystonia is a user drug-induced threatening reaction that warrants further investigation on the metabolism of these drugs and the contributing phenotypes to this adverse reaction.

## Introduction

Over a century ago, Oppenheim reported a series of cases of abnormal muscle movements and introduced the term “dystonia” [1]. Several clinical presentations of dystonia have been documented since Oppenheim's original observations. Dystonia is now referred to when describing a movement disorder represented by muscular contractions occurring in either sustained or intermittent rhythms involving different parts of the body [2]. The diagnosis of acute dystonia has notably been a challenge in the emergency unit, especially in the absence of a definitive diagnostic test. An accurate and rapid diagnosis is required for proper management [3]. Management can often be challenging. In drug-induced dystonia, the management relies on sedatives such as diazepam. Benzodiazepines are commonly used, however other agents with high anticholinergic activity, most notably diphenhydramine can also be used for symptomatic relief [4]. Despite its widespread usage in the management of drug-induced dystonic reactions, diphenhydramine has been recognized as a contributor to dystonia and dyskinesia in rare cases [5]. Diphenhydramine, an antagonist of the histamine H1-receptor, has a major adverse effect of central nervous system depression, reported in 50% of patients [6]. Paradoxically, some patients may have an excitatory response resulting in acute dystonic reactions [7]. Drug-induced dystonia is reported in a limited number of cases in the literature. Rarely, diphenhydramine was found to be the culprit. Our case describes a young female with a dystonic reaction secondary to the administration of diphenhydramine.

## Patient and observation

**Patient information:**a 25-year-old female patient, with no previous medical or surgical history or any documented drug or food allergies, presented to the emergency Unit for acute periorbital and perioral edema associated with pruritic body rash. The patient denies any particular family history. Her vitals upon presentation were within the normal range with a blood pressure of 139/104 mmHg, heart rate of 94 bpm, temperature of 37.3°C, and oxygen saturation of 99%. She was diagnosed with an allergic reaction, and she was administered a 25 mg dose of intravenous diphenhydramine as treatment.

**Clinical findings:** within 2 minutes of drug administration, the patient rapidly developed diminished responsiveness, generalized muscle spasticity, involuntarily forced jaw closure, involuntary tremors of the extremities, and involuntary orofacial movements. After this episode, her vital signs were heart rate of 101 bpm, blood pressure of 127/72 mmHg, and oxygen saturation of 97%. No strider or bronchospasm was noted. The patient was diagnosed as having an acute dystonic reaction.

**Diagnostic assessment:** a neurology consultation was requested in the emergency. Upon further investigation, the patient denied any history of recent head or neck trauma, substance abuse, or infection. She had no evidence of any chronic neurological disorder nor a family history of the latter. On neurologic examination, the patient was conscious and responsive to verbal stimuli. No focal deficit was noted, cranial nerve exam was intact, and her pupils were equal and reactive. Laboratory tests including complete blood count, creatinine, electrolytes, and liver function tests were ordered. They showed no abnormalities.

**Therapeutic intervention:** the patient was given a dose of 5mg of intravenous diazepam. She was admitted to the hospital for overnight monitoring. Her symptoms subsided after 2 hours. Her hospital stay was uneventful and she was discharged the next day.

**Follow up and outcomes:** the patient was advised to avoid drug preparations that contain diphenhydramine in the future. The diagnosis of diphenhydramine-induced acute dystonic reaction was made.

**Patient consent:** the patient gave her consent on clinical information to be reported.

## Discussion

The case we described is an atypical case of dystonia that we considered to be caused by the administration of diphenhydramine. In previously reported cases of acute dystonic drug reactions, onset was usually rapid, developing shortly after taking the antihistamine. Patients characteristically developed facial dystonia including trismus, dysarthria, and motor incoordination [4]. Their symptoms gradually resolved. This was similar to our patient. Symptoms started immediately after the injection, with orofacial movements, and extremities dystonia. Her symptoms resolved after 2 hours. However, in patients with documented long-term antihistamine use, resolution of symptoms was delayed [8,9]. Cases previously reported about drug-induced dystonia mention different classes of drugs in origin, but very few mention diphenhydramine [4]. In a case report written by de Leon *et al*., they described three cases of cytochrome P450 2D6 ultra metabolizes (CYP 2D6 UM) who all were previously healthy and had experienced paradoxical excitation after acute administration of diphenhydramine. They hypothesized that CYP450 2D6 UM had rapidly converted the administered diphenhydramine into compounds that cause excitation [8]. A phenotype study could be conducted in our patient to assess whether the CYP 2D6 UM is the cause.

## Conclusion

According to the literature, many drugs have been linked to acute dystonic reactions. Diphenhydramine, a usual go-to treatment in such cases can paradoxically be a trigger of acute dystonia, which can be unanticipated, life-threatening, and requires medical attention especially since this drug is readily available over-the-counter. This case highlights the need for further investigation in diphenhydramine metabolism and the link between diphenhydramine response and the CYP2D6 UM phenotype which could explain this unusual manifestation after diphenhydramine administration.
